# Development and Validation of an Oral Health Literacy Measurement for Primary School Children in Thailand

**DOI:** 10.1155/2022/9161619

**Published:** 2022-03-07

**Authors:** Pinpinut Wanichsaithong, Piyada Prasertsom

**Affiliations:** ^1^Division of Community Dentistry, Faculty of Dentistry, Chiang Mai University, Chiang Mai 50200, Thailand; ^2^Bureau of Dental Health, Department of Health, Ministry of Public Health, Nonthaburi 11000, Thailand

## Abstract

**Background:**

Oral health promotion programs have been implemented in primary schools for many years in Thailand. Oral health literacy has been introduced as a health promotion outcome; however, no assessment tool has been developed for this age group. The objective of this study was to develop and validate the Test of Functional Health Literacy in Dentistry for Primary School Children (P-TOFHLiD)*. Materials and Methods*. The P-TOFHLiD was developed by modifying contents and outlines using a previously validated tool for older adults, then verified by two experts for face validity. A cross-sectional study was conducted with samples collected from 118 grade-six students from two government schools in Chiang Mai Province, Thailand. The P-TOFHLiD and a previously validated word recognition test were administered, followed by oral examination to assess reliability, concurrent validity, convergent validity, and predictive validity and establish the cut-off score of the tool. The statistical analysis was performed using SPSS version 25.

**Results:**

The internal reliability of P-TOFHLiD was good (*α* = 0.808). The correlation coefficient between the P-TOFHLiD and grade point average was 0.478 (p value <0.001), which is the represented concurrent validity of the tool. Coefficients between P-TOFHLiD and a word recognition test was 0.422 (*p*-value <0.001) for convergent validity. P-TOFHLiD was significantly correlated with the number of missing teeth from tooth decay (*p*-value <0.05), but the correlation coefficient was poor (*r* = −0.100). The cut-off scores for adequate oral health literacy were set at ≥21 out of the total scores of 26.

**Conclusion:**

P-TOFHLiD presented good validity and reliability and was ready to use for oral health promotion program evaluation. However, the predictive validity of the P-TOFHLiD in predicting oral health status was questionable only.

## 1. Introduction

Thailand school health promotion programs started with the adoption of the World Health Organization's Global School Initiative concept [1]. The concept of health-promoting school policy has been proposed and implemented successfully in Thai primary schools as a starting point for building desirable healthy behaviors in children, with the cooperation of different sectors and factors [2]. Bureau of Dental Health, Ministry of Public Health, Thailand, has been working with primary schools by establishing the national oral health policy for children and has been conducting school-based oral health promotion programs since 2002 [3]. The oral health promotion programs in Thai primary schools involve teacher-supervised tooth brushing with fluoride toothpaste, oral health education, self-care practices regarding oral hygiene care and nutrition, and annual oral screening by dental professionals [4]. These interventions aim to improve the oral health of children by managing socio-environmental factors for students and expanding the work through the engagement of family and community, as well as at the national level. The expected outcomes of this program are to improve oral health outcomes, for example, reducing dental caries, enhancing good oral health behaviours, and setting good socio-environmental factors [5]. Oral hygiene education plays a pivotal role in reducing the incidence of dental caries; however, it has been debated that previous oral health promotion evaluations did not use a holistic approach and focused on clinical and behavioral changes rather than the wide range of strategies based upon the Ottawa Charter. There has been a need to develop evaluation methods that reflect the nature of oral health promotion practice [6].

Health literacy (HL) was introduced as one of the health promotion outcomes [7]. It was defined as “the personal, cognitive, and social skills that determine the ability of individuals to gain access to, understand, and use the information to promote and maintain good health” [8]. Functional health literacy is a fundamental level of three levels of HL: basic/functional, communicative/interactive, and critical HL. A person with adequate functional HL should have basic skills of reading and writing to function effectively in everyday circumstances [8]. Achieving critical HL can lead to personal empowerment in dealing with personal management of health's social determinants using comprehensive knowledge and skills [9]. As a result, different HL assessments have been developed recently and recommended for use as health outcome evaluations in health promotion [10].

Oral Health Literacy (OHL) is gaining attention in dentistry, especially since it was included in the Healthy People 2010 agenda for oral health promotion and disease prevention in the United States, referring to OHL as an important tool to reduce oral health disparities [11]. The concept of OHL has evolved according to the knowledge and concepts of general health literacy; however, the content of oral health is more complex and requires skills distinct from those of general health as well as differences in social, cultural, and linguistic contexts [12]. OHL has been suggested as one of the modern oral health promotion outcomes [6]. Measuring OHL has become a popular method for evaluating various oral health programs. Therefore, many OHL assessment tools have been developed and modified based on previous HL tools since 2007 [13].

In Thailand, several OHL measurements have been validated in the Thai language, with most tools modified from the original English versions, for example, ThREALD-30 [14], HeLD-Th [15], and OA-TOFHLiD [16], and were verified for adults and older adults. It was observed that in a study constructing a tool to measure parents' OHL associated with children's oral health [17], there was no instrument for directly measuring the OHL of primary school children. It was necessary to create an OHL tool to evaluate the outcomes of the Thai school oral health promotion program. Therefore, the objectives of this study were to develop a tool to measure the oral health literacy of school-age children and to assess the validity and reliability of the tool.

## 2. Materials and Methods

### 2.1. Development of a Tool

The Test of Functional Health Literacy in Dentistry for Primary School Children (P-TOFHLiD) was modified and developed from the previously validated tool for Thai older adults called the Test of Functional Health Literacy in Dentistry for Older Adults (OA-TOFHLiD) [16], which itself used the outline approach from the Test of Functional Health Literacy in Dentistry (TOFHLiD), originating from US researchers [18]. The original OA-TOFHLiD is divided into two sections: reading comprehension and prompts. There are four subtopics in the reading comprehension section: dental decay, gum disease, oral health prevention, and informed permission for tooth extraction. The prompt section is included 2 pictures of oral health product labels (toothpaste and chlorhexidine mouth rinse) with 4 questions for each label. The total scores are 39 for the reading comprehension section and 9 for the prompt section. The total scores of OA-TOFHLiD are 48, with cu off scores equal to/or more than 41, defined as indicating “*adequate oral health literacy*.”

To adapt OA-TOFHLiD for children in primary school-aged 11–14 years old, the difficulty level of the questionnaire had to be adjusted to match the children's levels of language learning and comprehension. The researcher and 2 experts (dental public health and health education) considered that children at this age should have basic knowledge of oral health, including all of these 4 topics: (1) oral organs and functions, (2) tooth decay, (3) oral hygiene care, and (4) food that causes tooth decay. Media and information accessible for this age range were reviewed, such as health and physical education textbooks from grades 1 to 6, as well as other oral health teaching materials discovered on the internet, to construct the Test of Functional Health Literacy in Dentistry for Primary School Children (P-TOFHLiD) content of approximately 1 paragraph (up to 200 words) for each topic. Then, the reading comprehension test was developed using the modified cloze approach [19], in which, four passages had a blank word every five to ten words. For each blank, there are four options with only one valid response. In total, the P-TOFHLiD consisted 4 parts of reading comprehension and the total scores of P-TOFHLiD was 26.

### 2.2. Validation of the Test in a Pilot Study

#### 2.2.1. Subject Recruitment and Data Collection

This study was a part of the project “Evaluation of Oral Health Promotion and Prevention Programs in schools,” for which the protocol was approved by the ethics committee of the Department of Health, Ministry of Public Health, Thailand (Ref. No. 381).

Since the prevalence of adequate or inadequate OHL was unknown in this population, sample size calculation could not be directly conducted. However, the sample estimation was performed according to the method recommended by Green (1991) [20]. He proposed *N* ≥ 50 + 8*m* (where *m* stands for the number of predictors in the model). In this study, the number of predictors was eight (see Table 1).  Therefore, it required at least 114 samples to achieve a medium-size relationship between the independent variables and the dependent variable.

A cross-sectional single visit study was conducted from December 2019 to January 2020. Purposive sampling was used to select two different primary schools to include subjects with diverse socio-cultural backgrounds in Chiang Mai, Thailand. Two classrooms of Grade 6 were randomly selected from each school, including 41 children from Wat Suan Dok Municipal School (Mueang District; Urban), and 77 children from Wat Weruwan School (Saraphi District; Suburban). The total number of children who participated in this study was 118. One week before data collection at the selected schools, a study information sheet and a written informed consent form were sent to all the parents to obtain their consent for the child to participate in the study. Inclusion criteria were children who were able to read and write Thai, and parents permitted their children to participate in the study. Exclusion criterion was children with intellectual or learning disabilities that could bias their functional OHL (reading and writing texts).

On the data collection day, children were asked to fill out two questionnaires, including general background information and P-TOFHLiD. A dental examination was performed by a qualified dentist (Cronbach's alpha = 0.90). The WHO's community oral health survey was used, and the DMFT index (included in the survey), was applied [21]. Finally, children performed the previously validated OHL test as a reference tool, which is a pronunciation of 30 dental words (ThREALD-30) [14]. The overall process took approximately 30 minutes to 1 hour.

#### 2.2.2. Data Analysis

The statistical analysis was conducted using the SPSS program version 25 for Mac (Armonk, NY, USA). General information was presented by descriptive statistics (maximum, minimum, mean, and percent). Psychometric properties were tested to determine whether the newly developed tool had appropriate validity and reliability. The study validation process was according to the previous OHL validation studies [16] and guidelines. The assessment included;

*(1) Internal Reliability*. The internal reliability of the P-TOFHLiD was analyzed using Kuder-Richardson's test, which investigated the correlations between different items on the same tool. The acceptable result was that Cronbach's alpha was greater than 0.7 [22].

*(2) Concurrent Validity*. To examine concurrent validity, we examined whether the OHL scores obtained from this tool were able to distinguish between groups that should be theoretically different. Hypotheses were that higher age, higher grade point average (G.P.A.), and higher daily stipends, would be correlated with higher OHL scores, assessed by Spearman's correlation (*p*-value <0.05).

*(3) Convergent Validity*. This validity refers to the concept that the new tool should be similar to the previously validated tools that measure the same phenomenon. Therefore, we examine whether the scores from the newly produced tool (P-TOFHLiD) were correlated with and in the same direction as the validated measurement (ThREALD-30) assessed by Spearman's correlation (*p*-value <0.05).

*(4) Predictive Validity*. This property tested the ability of OHL scores to predict the possible future consequences. It was hypothesized that low OHL scores should be theoretically associated with the occurrence of poor oral health outcomes, for example, tooth decay, tooth extraction, or poor oral health behaviour. This study assessed the predictive validity of the OHL scores obtained from P-TOFHLiD by using Spearman's correlation (*p*-value <0.05) for predicting continuous variables and the Mann-Whitney *U* test for predicting dichotomous variables.

In addition, the “good oral health behaviour (code 1)” variable was created, which represents the combination of four characteristics: (1) utilisation of dental services at least once in lifetime, (2) brushing their teeth at least twice a day, (3) eating snacks only some days, or infrequently, and (4) consuming sugar-sweetened beverages (SSBs) or soft drinks only some days or infrequently. One who failed to meet all four criteria was assigned to “poor oral health behaviour (Code 0).” This variable was assessed by the Mann-Whitney *U* test to compare OHL scores between good and poor oral health behaviour. To prevent consequences caused by multiple testing, especially Type I errors or a false positive, the Bonferroni correction [23] was conducted to adjust the probability by dividing the statistically significant *p*-value by the number of parameters to test (0.05/6). The adjusted significant *p*-value for the predictive validity was less than 0.008 for the Mann-Whitney *U* test.

*(5) Cut-Off Point Consideration*. Receiver operating characteristic (ROC) curve analysis was used to generate a cut-off score for the newly developed tool to differentiate adequate or inadequate OHL levels. The Area Under the Curve (AUC) was considered to evaluate the ROC curve's performance at a significant level of *p*-value less than 0.05. Cut-off scores were determined by maximizing the sensitivity (Se) and specificity [24] to predict associated oral health outcomes using Youden's index [25].

## 3. Results and Discussion

### 3.1. Characteristics of Participants

The total number of participants was 118. All the participants were students at primary schools (grade 6), aged in the range of 11–16 years old (mean 11.93 years old, SD 0.69). The grade point average of the participants was a range between 1.33 and 4.00 (mean 2.73, SD 0.70). The daily stipend that children receive from parents or caregivers was approximately 54 THB, SD 25.38 (1.6 USD), with a maximum of 200 THB and a minimum of 10 THB.

Table 2 presents the general characteristics of all the participants, categorized into several subgroups. The majority of participants were male (55.1%), living in suburban areas (65.3%), and having a mother and/or father as primary caregiver(s) (85.6%). Most of the participants had at least one active tooth decay that needed to be treated (71.2%) and did not have any tooth sealant or fillings (64.2%). Only one-third (31.4%) of the participants were classified as having good oral health behaviour.

### 3.2. Scores of P-TOFHLiD and Reference Measurement

Table 3 shows the P-TOFHLiD scores in each part, as well as the total P-TOFHLiD scores and the scores of reference measurement (ThREALD-30). The participants had the greatest mean scores in Part 1: Tooth structures and oral function (Mean 6.44, SD 0.95), and the lowest mean scores in Part 4: Food that causes tooth decay (Mean 3.27, SD 1.59). P-TOFHLiD and ThREALD-30 had mean total scores of 19.39 (SD 4.36) and 23.94 (6.13), respectively.

#### 3.2.1. Internal Reliability

P-TOFHLiD was assessed for internal agreement by using the Kuder Richardson 20 (KR-20) test, which determined that the correlation should not be less than 0.7. The result demonstrated that Cronbach's alpha was 0.808; therefore, P-TOFHLiD was considered to have very good internal reliability.

#### 3.2.2. Concurrent Validity

For categorical variables, males had significantly lower P-TOFHLiD scores than females, as shown in Table 2 (*p*-value <0.001). The results of Spearman's correlations between P-TOFHLiD scores and continuous variables are presented in Table 1. A higher GPA was found to be associated with a higher OHL score (*r* = 0.478, *p*-value <0.001). The reference tool ThREALD-30 also discovered this association (*r* = 0.339, *p*-value <0.001), but our newly constructed instrument had a greater correlation. There were no statistically significant correlations between OHL scores and other variables.

#### 3.2.3. Convergent Validity

From Spearman's correlation analysis, the findings in Table 1 revealed that OHL scores from P-TOFHLiD tended to correlate in the same direction as OHL scores from the reference instrument ThREALD-30, with a correlation of *r* = 0.422 (*p*-value < 0.001).

#### 3.2.4. Predictive Validity

This validity was described as the ability of OHL scores from the developed tool to predict possible future outcomes. The results showed that the P-TOFHLiD scores were negatively correlated with the number of tooth losses from decay (missing teeth; M). The resulting correlation (*r*) was −0.100 (*p*-value = 0.047).

Table 2 presents the additional results from comparing the P-TOFHLiD scores between dichotomous variables. It was observed that children with good oral behaviour had significantly better OHL scores than those with poor oral health behaviour (*p*-value = 0.006).

#### 3.2.5. Cut-Off Scores Consideration

**Figure 1:**
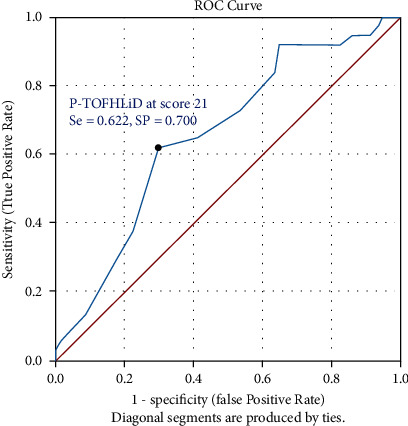
Receiving operation characteristic (ROC) curve of P-TOFHLiD scores to predict good oral health behaviour for considering appropriate cut-off scores.

To propose appropriate cut-off scores for the newly developed test, ROC curve analysis is demonstrated in Figure 1. The Area Under the Curve (AUC) was 0.657 (*p*-value = 0.007) which was an acceptable level. The results found that with a score of 21 on the P-TOFHLiD, Se and Sp were 0.622 and 0.700, respectively, which were judged acceptable.

In comparison with other cut-off scores, the total sum of Se and Sp at P-TOFHLiD at score 21 was 1.322, which was the greatest sum. As a result, the recommended P-TOFHLiD cut-off scores were “greater than or equal to twenty-one (≥21)” as an adequate OHL, and “less than twenty-one (<21)” as an inadequate OHL.

## 4. Discussion

OHL has never been directly evaluated in primary school-age children, while the increasing importance of this group combined with their high degree of heterogeneity and poorly understood risk factors means such work is needed. The previous review found that there was no validated OHL tool specifically for this age group [13]. Measurements of OHL were popular among adults because there is an idea that children in primary school have not yet fully developed cognitive skills. It usually takes around 16 years for intellectual development and cognitive skills to reach adult-level maturity [26]. Furthermore, it is considered that children's oral health is the responsibility of their parents, as kids may not be capable of caring for their oral health [27]. As a result, the previous studies presented the use of adult OHL tools to assess parents' or caregivers' OHL and its associations with children's health, for example, dental caries [28–30], oral health behaviour [31, 32], dental expenditures [31], and oral health-related quality of life [33].

The objective of this study was to develop a tool to assess OHL in primary school children aged 11 to 14, the age range in which children usually take responsibility for personal care. Most parents allow their children to do basic daily activities such as personal hygiene care (brushing teeth), making decisions about eating meals or purchasing snacks during school hours and reporting an abnormality or a symptom related to the mouth and teeth. Children require functional OHL to act effectively and independently in various daily routines. Additionally, Thai children have gained knowledge about health, including oral health, in the curriculum of health education and physical education since grade one in primary school. Therefore, the functional OHL tool that we developed in this study, which aims to test basic knowledge and reading comprehension skills related to oral health, is appropriate for children of this age group and suitable for health promotion program evaluation. Based on the previous systematic review, this type of instrument does not measure skills that exceed children's abilities according to their cognitive development [34].

Face validity was measured by the appropriateness of the test's content and how proper language was used for children. The P-TOFHLiD demonstrated satisfactory face validity because the participants could complete the questionnaire on their own within seven to fifteen minutes. The questionnaire contains four subtopics, which were arranged from the easy part (tooth structures and oral functions) and gradually increased the level of difficulty to the final subtopic (food that causes tooth decay) to encourage children to complete the test by starting with the easy one, but distinguishing individuals with adequate or inadequate OHL by the final part. The mean score of each subtopic is presented according to our hypothesis that the last part should show the lowest mean. The P-TOFHLiD will be beneficial to health professionals and schoolteachers for utilising the tool to assess children's OHL and deliver an appropriate intervention emphasizing the hardest subgroup. The previous study suggested that implementing dental-health education together with creating healthy behaviours was one of the most effective health promotion programs to prevent dental caries in children [35].

The results of this study supported concurrent validity for P-TOFHLiD. The P-TOFHLiD and ThREALD-30 scores were significantly correlated with grade point average or GPA. However, when compared with the reference instrument, the P-TOFHLiD scores had a stronger correlation with GPA. It might be because P-TOFHLiD requires a range of skills and abilities to complete, including health knowledge, reading, language comprehension, and numeracy. Therefore, the OHL assessment might be linked to a GPA evaluation, which measures an individual's overall success in formal schooling. This finding corresponded to the previous study in Thai college students that observed a lower GPA was associated with lower HL scores [36]. WHO also suggested that enhancing HL in children and young people could have an impact on improving academic achievement [37].

The convergent validity of the P-TOFHLiD was confirmed by the positive correlation with the ThREALD-30, which was previously validated in the Thai population [14]. Although the correlation between P-TOFHLiD and ThREALD-30 was slightly low (*r* = 0.422), a significant *p*-value was observed (*p*-value < 0.001). It might be explained by the fact that the newly produced tool (reading comprehension) and reference tool (word recognition) characteristics were not fully linked. However, because they were designed to test the same level of OHL (functional oral health literacy), it was acceptable to use them in this study. In addition, the original TOFHLiD was previously validated using the word recognition test REALD-99 to determine convergent validity; nevertheless, the original TOFHLiD and REALD-99 had a greater correlation (*r* = 0.82, *p*-value 0.05) [18]. Therefore, in a validation study, it would be preferable to employ a long version of the word recognition test (REALD-99) as a reference tool.

To confirm the predictive validity of the P-TOFHLiD, it was anticipated that OHL might be associated with oral health status and oral hygiene behaviour. The results demonstrated that P-TOFHLiD had a significant ability in predicting oral health status compared with the reference tool. The previous parental OHL study also suggested that a reading comprehension instrument had a stronger association with children's oral health status than a word recognition instrument [38]. However, the findings from this research partially supported our hypothesis. Rather than the overall DMFT, only the number of missing teeth from decay (M) was associated with P-TOFHLiD, unlike the previous studies of the original tool in older adults [16, 39]. That may be because, throughout childhood, children's oral health has been primarily under the supervision of a caregiver or parent. In addition, Thailand has a strong health policy and concrete indicators to promote oral health services for primary school children. For example, in urban as well as rural areas, primary school children in grade six should receive free dental services such as dental check-ups, dental sealants, or dental health education at least once a year [4]. Consequently, the children's OHL may not have a significant effect on their oral health status at this stage. Children's OHL, on the other hand, was found to have a substantial influence on their oral hygiene habits. Children with better oral hygiene had significantly higher OHL scores in this research. This finding was consistent with a prior study in Japan, that found that school-aged kids with higher HL scores brush their teeth cleaner than those with lower HL scores [40]. As a result, we opted to use the OHL scores to predict the variable “good oral health behaviour” instead of “good oral health status” in the ROC curve to create the appropriate P-TOFHLiD cut-off scores.

For the future objectives, the use of oscillating and sonic toothbrushes to reduce the bacterial load and the use of biomimetic hydroxyapatite-based toothpaste to have a proactive action to reduce the incidence of caries [41, 42] will be beneficial in caries prevention in primary school children.

## 5. Conclusions

This study focused on the development of a new OHL instrument for primary school children. The P-TOFHLiD presented good validity and reliability and was ready to use for oral health promotion program evaluation. However, the predictive validity of the P-TOFHLiD in predicting oral health status was questionable. It may need to be reassessed with a larger sample size in a future study.

## Figures and Tables

**Table 1:** Correlations of the P-TOFHLiD with variables for different validity assessments.

Variables	Newly developed tool P-TOFHLiD		Reference tool ThREALD-30	
	*R*	*p*-Value	*r*	*p*-Value
Concurrent validity				
Age	**0.023**	**0.805**	−**0.082**	**0.377**
Grade point average	**0.478**	**<0.001** ^*∗∗*^	**0.339**	**<0.001** ^*∗∗*^
Daily stipend	−**0.090**	**0.335**	−**0.061**	**0.515**
Convergent validity				
ThREALD-30	0.422	**<0.001** ^*∗∗*^	—	—
Predictive validity				
DMFT (overall)	0.009	0.921	−0.010	0.916
Number of decay	−0.067	0.286	−0.073	0.434
Number of missing	−0.100	**0.047** ^*∗*^	0.040	0.665
Number of filled	0.184	0.342	0.127	0.173

Significant value: ^*∗*^*p*-value < 0.05, ^*∗∗*^*p*-value < 0.001.

**Table 2:** Characteristics of study participants and comparing P-TOFHLiD scores among different dichotomous characteristics.

Variables	Characteristics	*N*	Percent	P-TOFHLiD scores (total 26)		
				Median	Interquartile range	*p*-value^Ψ^ (2-tailed)
Gender	Male	65	55.1	19.00	14.25–21.00	**<0.001** ^*∗*^
	Female	53	44.9	22.00	20.00–23.00	

School	Urban	40	34.7	21.50	19.00–23.00	0.059
	Suburban	77	65.3	20.00	16.00–22.00	

Primary caregiver	Mother and/or father	101	85.6	21.00	16.50–23.00	0.711
	Others	17	14.4	19.50	19.00–21.75	

Having at least one active tooth decay^*δ*^	No	33	28.0	20.00	16.00–23.00	0.947
	Yes	84	71.2	20.00	18.00–22.75	

Having at least one tooth sealant or filling^*δ*^	No	76	64.4	20.00	16.00–22.00	0.055
	Yes	41	37.5	22.00	18.50–23.00	

Having good oral health behaviour^*δ*^	No	80	67.8	20.00	16.00–22.00	**0.006** ^*∗*^
	Yes	37	31.4	22.00	19.00–23.00	

^*δ*^There was missing data in the group. ^Ψ^*p*-value obtained from the Mann-Whitney *U* test. ^*∗*^*p*-value less than 0.008.

**Table 3:** Characteristics of OHL scores obtain from the P-TOFHLiD and the TH-REALD.

Measure	Part	Min	Max	Mean (S.D.)	Scores at Percentile50
P-TOFHLiD	1. Tooth structures and functions (7)	1	7	6.44 (0.95)	7.00
	2. Basic knowledge about signs and symptoms tooth decay (6)	1	6	4.68 (1.28)	5.00
	3. Oral hygiene care (7)	0	7	5.00 (1.64)	5.00
	4. Foods that cause tooth decay (6)	0	6	3.27 (1.59)	3.00
	Total scores (26)	5	26	19.39 (4.36)	20.00

ThREALD-30	Total scores (30)	2	30	23.94 (6.13)	26.00

## Data Availability

The validation data and the P-TOFHLiD tool used to support the findings of this study are available from the corresponding author upon request.
